# Neural graphs: small-worlds, after all?

**DOI:** 10.1186/1471-2202-15-S1-O13

**Published:** 2014-07-21

**Authors:** Michelle Rudolph-Lilith, Lyle E Muller

**Affiliations:** 1 Unité des Neurosciences, Information et Complexité (UNIC), CNRS UPR-3293, Gif-sur-Yvette, 91198, France

## 

In recent years, small-world graphs have gained considerable interest as models of real-world systems, which often display features residing between regularity and randomness. The most notable of these models is the Watts-Strogatz graph [1], though alternatives have been proposed [2]. The unifying characteristics of these models are that any two nodes are joined with a small number of links between them (i.e. short path length), while at the same time connected node pairs exhibit an abundance of triangular relations resulting in a high degree of local redundancy (i.e. high clustering).

Theoretical investigations of small-world graph models have generally applied asymptotic evaluations in the limit of large system size [3] or the continuum approximation [4] to the algorithmic definition of the graph, in the absence of an analytic representation. In this study, we introduce a generative model of directed small-world graphs, a canonical model of Watts-Strogatz digraphs, and propose an approach that yields the graph’s defining adjacency matrix in algebraic terms, with the goal to provide mathematically rigorous access to the study of finite-size small-world graphs [5]. The proposed approach makes use of random annihilation operators whose algebraic properties can be utilized to assess algebraically well-defined graph-theoretic measures in an analytically exact framework, valid nonasymptotically for all graph sizes. We demonstrated the application of our approach by calculating, for the first time, the asymmetry index and total clustering coefficient of small worlds in an exact fashion.

We then utilize the exact nonasymptotic expression for the clustering coefficient in order to assess the small-worldness of structural brain networks in an analytic setting. Using the number of nodes and edges of the given brain networks to construct the equivalent small-world network, we observe that a significant edge rewiring of at least 20% up to 60% is required to produce the small-worldness indices observed in these networks. Importantly, the maximum of the small-worldness index however occurs in all cases at one order of magnitude lower than the required rewiring found. This result suggests that neural graphs reside far away from the small-world regime of the Watts-Strogatz model.

## References

[B1] Watts DJ Strogatz SH Collective dynamics of ‘small world’ networks Nature 1998 15 440 10.1038/30918 9623998

[B2] Monasson R Diffusion, localization and dispersion relations on “small-world” lattices Eur Phys J B 1999 15 555 10.1007/s100510051038

[B3] Barrat A Weigt M On the properties of small-world network models Eur Phys J B 2000 15 547 10.1007/s100510050067

[B4] Newman MEJ Moore C Watts DJ Mean-field solution of the small-world network model Phys Rev Lett 2000 15 3201 10.1103/PhysRevLett.84.3201 11019047

[B5] Rudolph-Lilith M Muller LE Algebraic approach to small-world network models Phys Rev E Stat Nonlin Soft Matter Phys 2014 15 1 012812 10.1103/PhysRevE.89.012812 24580286

